# 3D Printing of Glass Micro-Optics with Subwavelength Features on Optical Fiber Tips

**DOI:** 10.1021/acsnano.3c11030

**Published:** 2024-03-29

**Authors:** Lee-Lun Lai, Po-Han Huang, Göran Stemme, Frank Niklaus, Kristinn B. Gylfason

**Affiliations:** † Division of Micro and Nanosystems, School of Electrical Engineering and Computer Science, KTH Royal Institute of Technology, Stockholm 10044, Sweden

**Keywords:** direct laser writing, microstructured fiber, 3D glass, optical fiber sensing, polarization beam splitter

## Abstract

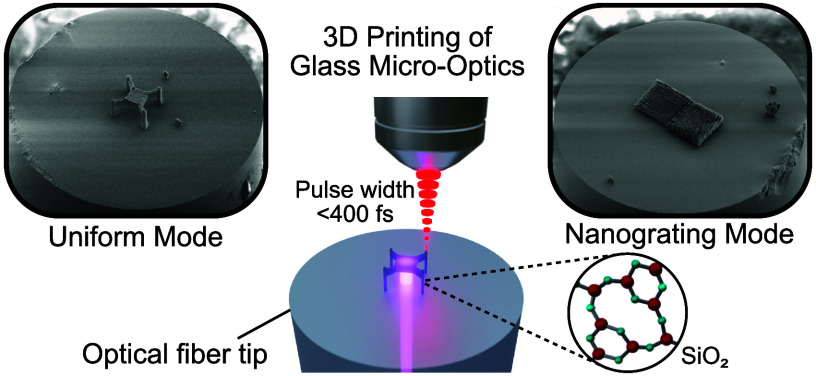

Integration of functional materials and structures on the tips of optical fibers has enabled various applications in micro-optics, such as sensing, imaging, and optical trapping. Direct laser writing is a 3D printing technology that holds promise for fabricating advanced micro-optical structures on fiber tips. To date, material selection has been limited to organic polymer-based photoresists because existing methods for 3D direct laser writing of inorganic materials involve high-temperature processing that is not compatible with optical fibers. However, organic polymers do not feature stability and transparency comparable to those of inorganic glasses. Herein, we demonstrate 3D direct laser writing of inorganic glass with a subwavelength resolution on optical fiber tips. We show two distinct printing modes that enable the printing of solid silica glass structures (“Uniform Mode”) and self-organized subwavelength gratings (“Nanograting Mode”), respectively. We illustrate the utility of our approach by printing two functional devices: (1) a refractive index sensor that can measure the indices of binary mixtures of acetone and methanol at near-infrared wavelengths and (2) a compact polarization beam splitter for polarization control and beam steering in an all-in-fiber system. By combining the superior material properties of glass with the plug-and-play nature of optical fibers, this approach enables promising applications in fields such as fiber sensing, optical microelectromechanical systems (MEMS), and quantum photonics.

In recent decades, integration of functional materials and structures on the tips of optical fibers has created opportunities for a range of applications in sensing,^1,2^ imaging,^3^ and optical trapping.^4,5^ The optical fiber tip provides an inherently light-coupled platform that allows for the interaction of the light guided by the fiber core with the device integrated onto the tip.^6^ Moreover, fiber-tip devices typically have a small footprint, low insertion loss, and exceptional compatibility with standard optoelectronic components due to the plug-and-play nature of optical fiber technology. However, the small area and delicate nature of the optical fiber tip make it challenging to apply standard microfabrication processes optimized for planar substrates. To overcome these challenges, researchers have proposed several approaches that are tailored for fabrication on fiber tips, such as chemical etching,^7,8^ electron-beam lithography,^9,10^ focused ion-beam milling,^4,11^ nanoimprint lithography,^12,13^ self-guiding photopolymerization,^14^ 3D μ-printing technology,^15^ and 3D direct laser writing (DLW).^16,17^ Among these approaches, 3D DLW is particularly attractive because it enables the fabrication of arbitrary 3D structures with submicrometer resolution and complex designs. A plethora of intricate fiber-tip microstructures has been manufactured using the 3D DLW technique, and applications in beam shaping and sensing have been demonstrated.^18−21^ However, these fiber-tip devices were made of polymers, which are known to swell and deform upon exposure to many organic solvents. This greatly restricts the potential applications of polymer-based devices, especially in sensing, where high levels of accuracy and repeatability are required. Moreover, the glass transition temperature of most polymers is below 300 °C,^22^ making them susceptible to deformation and failure at high temperatures. Additionally, polymers tend to degrade over time, further limiting their applicability for long-term use and harsh environments.

Unlike polymers, inorganic glasses are favorable materials for fiber-tip devices, as they show excellent chemical resistance, thermal stability, hardness, and optical transparency across a broad range of wavelengths. Although 3D printing of inorganic glass structures with submicrometer resolution has been demonstrated,^23,24^ these approaches required thermal treatment at elevated temperatures above 650 °C for more than 12 h, making them challenging to apply to optical fibers that comprise temperature-sensitive coatings and jackets. A recent work employs a conversion process under deep ultraviolet (DUV) and ozone exposure that enables the 3D printing of silica glass with a low-temperature DUV treatment at 220 °C.^25^ However, this temperature is still higher than the temperature limit of standard telecom fibers, and the ozone treatment would degrade the coatings and jackets of the fibers. Recently, we reported two techniques that enable 3D printing of uniform silica glass and silicon-rich (Si-rich) glass nanogratings by DLW in hydrogen silsesquioxane (HSQ) without thermal treatment.^26,27^ Although a silica-glass structure on a fiber tip was shown in our prior work,^26^ this structure did not have any functionality and relevant applications were not investigated. Furthermore, the refractive index of the printed glass is not known. Thus, the promising avenue of integrating functional glass micro-optics directly on fiber tips remained unexplored.

In this work, we demonstrate 3D printing of inorganic glass micro-optics with submicrometer resolution on standard SMF-28 fiber tips using femtosecond laser pulses with a wavelength of 1040 nm to selectively cure HSQ on the optical fiber tip. Based on the two curing modes reported in our previous studies,^26,27^ we demonstrate two devices: a fiber-tip refractive index sensor and an ultracompact polarization beam splitter (PBS). The fiber-tip refractive index sensor, benefiting from the exceptional chemical stability of silica glass, allowed us to measure the index of aggressive organic solvents. The ultracompact PBS enabled polarization control and beam steering through the subwavelength gratings printed on the fiber tip, which has the potential to replace discrete optical components that are bulky and difficult to align. The ability to fabricate free-form glass micro-optics on optical fiber tips using our 3D printing technique will enable a wide range of applications.

## Results and Discussion

### 3D Printing of Glass on the Optical Fiber Tip

**Figure 1:**
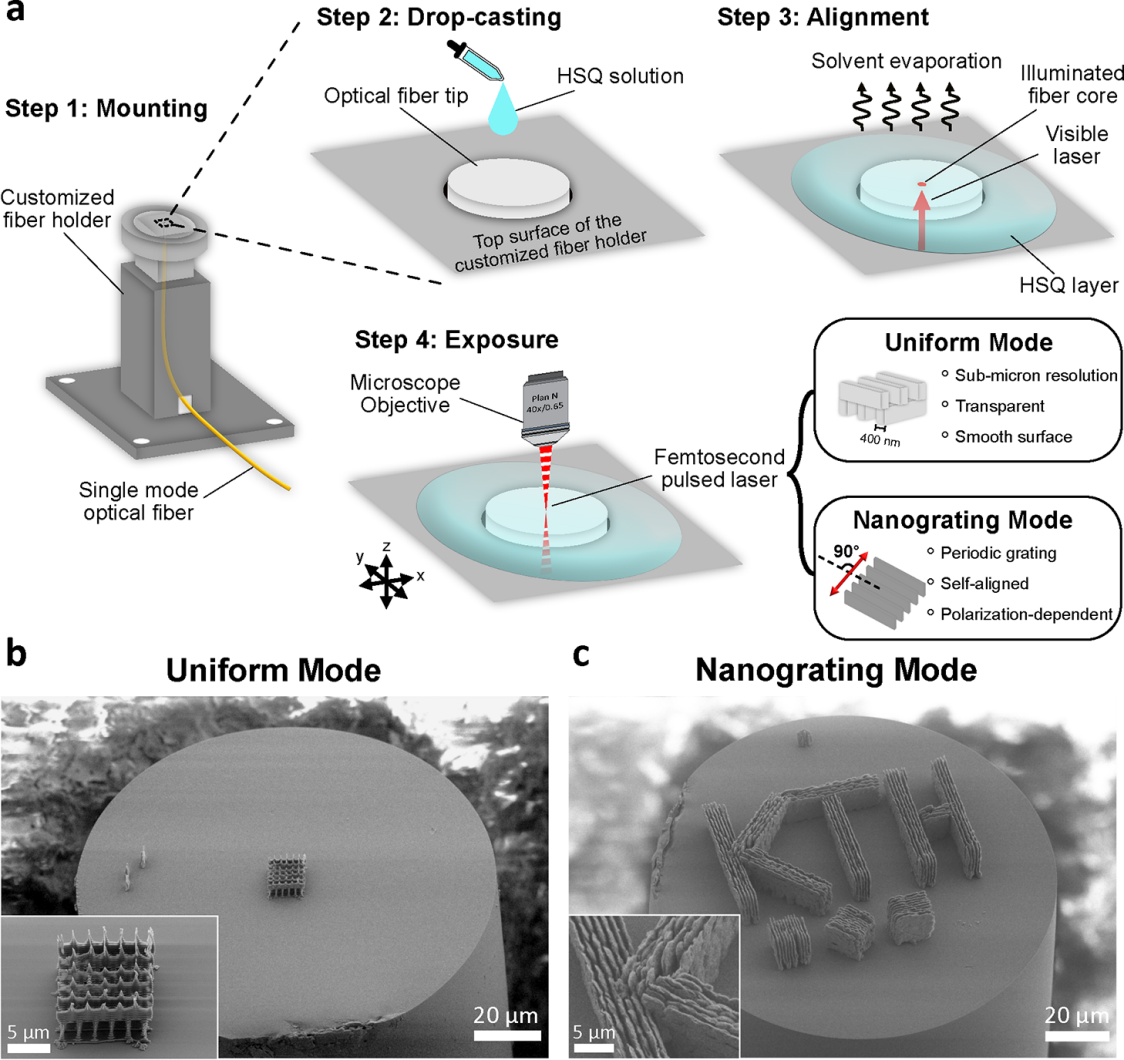
Printing process and example 3D structures in glass on optical fiber tips. (a) The fabrication process. Step 1: Mounting single-mode optical fiber in a customized fiber holder. Step 2: Drop-casting HSQ solution on the optical fiber tip. Step 3: Evaporating solvent. Injecting a visible laser from the other end of the fiber to illuminate the fiber core for alignment. Step 4: Exposing the HSQ layer with the femtosecond pulsed laser. Uniform Mode and Nanograting Mode can be selected by choice of exposure parameters. (b) A woodpile structure printed using Uniform Mode. The inset shows a close-up of the printed structure: the lateral width of each beam is below 400 nm. (c) Characters “KTH” and three blocks printed using Nanograting Mode. The inset shows that the three segments of the letter “K” are made of Nanogratings with distinct selected orientations.

The 3D printing of inorganic glass structures on optical fiber tips includes four steps, as shown in Figure 1a. In step 1, we cut a single-mode optical fiber to the desired length and then cleaved fiber facets on both ends. To print on the fiber tip, the fiber tip needs to be fixed firmly. Therefore, we machined a customized fiber holder into aluminum through which we threaded the optical fiber and then fixed the base to the motorized stage of our femtosecond laser workstation. In step 2, we drop-casted the HSQ solution onto the fiber tip, which formed a dome shape that covered the fiber tip. The HSQ solution is composed of HSQ powder dissolved in toluene with a concentration of 40 wt %. We used a higher concentration of HSQ compared to our previous work^26^ because it resulted in the viscosity required to grow a dome-shaped HSQ layer with a thickness of around 100 μm by drop-casting only two droplets of HSQ solution. See the Methods section for details of sample preparation. In step 3, we dried the HSQ by evaporating the solvent and leaving behind a thin hard layer covering the fiber tip. To align the fiber core for DLW, we injected 650 nm visible laser light from the other end of the fiber, illuminating the fiber core. In step 4, we used a femtosecond laser workstation for DLW. The laser operates at a 1040 nm central wavelength with a pulse width of less than 400 fs. We used a 40× microscope objective with a numerical aperture of 0.65 to focus the laser on the sample. The sample is fixed to a three-axis linear motorized stage, which allowed us to control the movement of the sample with an accuracy of 0.3 μm. The movement of the stage can be programmed by software. During DLW, the ultrashort laser pulses induce nonlinear absorption at the most intense central part of the laser focus and selectively cure the HSQ there, i.e., the HSQ within the laser focus absorbs multiple photons and is thereby cross-linked. On the contrary, the HSQ outside remains uncured and soluble by the developer.^26^ Finally, we employed a home-built development setup to remove the uncured HSQ and release the 3D-printed silica glass structure on the fiber tip. See the Methods section for details. After the development process, SEM inspection shows no visible HSQ residue remaining on the fiber tip.

To explore different applications, we exploited two printing modes from our previous studies,^26,27^ namely “Uniform Mode” and “Nanograting Mode.” Parts b and c of Figure 1 show the demonstrators printed using Uniform Mode and Nanograting Mode, respectively. We selected the printing mode by tuning the parameters of the femtosecond laser workstation. The details of each printing mode follow.

#### Uniform Mode

Uniform Mode allowed us to print solid silica-glass structures with submicrometer resolution, high optical transparency, and smooth surfaces. The typical single-pulse energy of the laser is 25–30 nJ, the repetition rate is 10 kHz, and the moving speed of the motorized stage is 0.5 μm/s. Using this writing speed, the smallest voxel dimensions we obtained were below 100 nm in width and 300 nm in height. The hardness and reduced elastic modulus of the as-printed structure measured 2.4 ± 0.2 GPa and 40 ± 2 GPa, respectively,^26^ which are one to two orders higher than that of polymers. Figure 1b shows a woodpile structure printed by using Uniform Mode, where the lateral width of each beam is below 400 nm.

**Figure 2:**
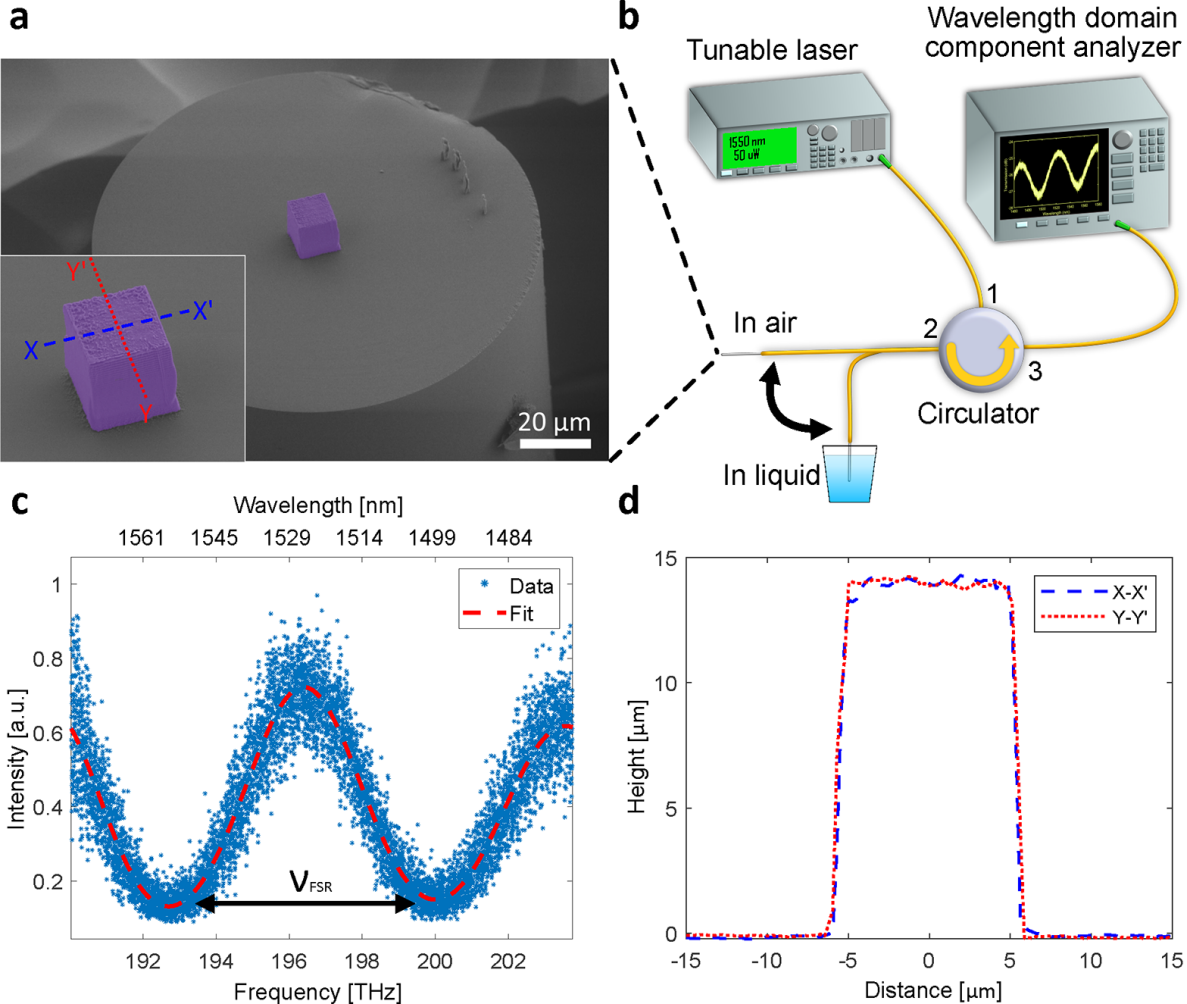
Characterization of the refractive index of a 3D-printed glass cube. (a) The colored SEM image shows the solid glass cube printed on the fiber tip. The inset is a close-up view of the cube. (b) The experimental setup for the refractive index characterization. (c) The reflection spectrum obtained from the fiber-tip glass cube immersed in a 50 wt % sucrose solution. (d) 2D profile plot shows the profile data measured by the optical profilometer. The plots were captured across the center of the cube, indicated by the dashed lines in (a).

Uniform Mode is well-suited for fabricating solid micro-optical components such as mirrors and lenses due to its capability to print transparent and smooth structures. Although the refractive index of the 3D-printed silica glass is a crucial parameter for micro-optical components, it has not been previously evaluated.^26^ Using the process developed in this work, we printed a solid glass cube on the fiber tip, which allowed us to measure the refractive index of the 3D-printed glass. Figure 2a shows a colored SEM image of the cube with a square base with 11 μm side length. Because the center of the cube is aligned with the fiber core and its lateral dimensions are larger than the mode field diameter of the fiber, the cube functions as a solid Fabry–Pérot etalon, where the light injected from the fiber circulates in the cube. The free spectral range (FSR) of a Fabry–Pérot etalon is
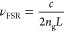
1where *c* is the speed of light in vacuum, *n*_g_ is the group refractive index of the glass in the printed cube, and *L* is the height of the cube. Using the experimental setup illustrated in Figure 2b, we measured the reflection spectrum of the cube in the optical telecommunication S, C, and L bands between 1470 and 1570 nm. To improve the accuracy of our refractive index determination, we collected multiple sets of reflection spectra with the cube placed in different environments, including air, deionized water, and sucrose solutions at different concentrations. Figure 2c shows the reflection spectrum obtained from the cube immersed in a 50 wt % sucrose solution. The experimental data are fitted with a sum of sines model for estimating the FSR (see Methods for details). Data measured in other environments are shown in the Supporting Information, Figure S1 and Table S1. To determine the height of the cube, we used an optical profilometer (see Methods for details), and the resulting profile data are shown in Figure 2d. The height of the cube measures 14.2 μm. Finally, we used eq 1 to compute the index of the cube using ν_FSR_ obtained from the reflection spectrum and *L* measured by the optical profilometer. The index of the 3D-printed glass cube is estimated to be 1.47 ± 0.01, agreeing well with the established reference group index of fused silica in this wavelength range,^28,29^ which is 1.4624 ± 0.0003 (see Methods for details).

#### Nanograting Mode

Nanograting Mode allows us to print self-aligned nanogratings in a Si-rich oxide glass with residual hydrogen (HnSiOx, *n* < 1 and *x* < 1.5).^27^ The typical single-pulse energy of the laser is 45–50 nJ, the repetition rate is 50 kHz, and the moving speed of the motorized stage is 500 μm/s. Each nanograting consists of multiple nanoplates that are equally separated and aligned to each other, with a grating periodicity of approximately 1.1 μm that is independent of the laser polarization or laser exposure dose.^27^ We attribute the grating periodicity to two main factors: (1) the wavelength of the writing laser and (2) the refractive index of the HSQ solution.^30^ Therefore, one can change the grating periodicity by printing with a laser with a different wavelength or by doping the HSQ solution with nanoparticles to modify its refractive index. The orientation of the nanogratings is polarization-dependent, as illustrated in the bottom right of Figure 1a, the orientation of the grating is perpendicular to the polarization of the laser beam indicated by the red double arrow. Therefore, we can control the orientation of the printed nanogratings by rotating the half-wave plate in a femtosecond laser workstation. Compared to structures printed using Uniform Mode, structures printed using Nanograting Mode have rougher surfaces, while the faster printing speed allows us to print larger structures. Our experiments indicate that the surface roughness of the printed structure could be reduced by employing a higher laser pulse energy during the writing process.^27^Figure 1c shows the characters “KTH” and three blocks with different orientations printed on a fiber tip using Nanograting Mode. The inset shows that the letter “K” comprises three sets of nanogratings with three different selected orientations. All of the grating nanoplates are continuous and well-aligned.

Different approaches that enable the fabrication of periodic nanostructures were proposed recently. For example, Braun et al. reported a technique for the fabrication of nanostructured arrays by DLW, followed by metal deposition and lift-off processes,^31^ and Zhu et al. presented self-organized optical metasurfaces by employing the resonant absorption of laser light.^32^ Compared to these methods, our work features the ability to realize more sophisticated structures, e.g., a nanograting array with subordinate periodic nanostructures that have different orientations thanks to the polarization dependency of the gratings. Moreover, our 3D printing technique enables the printing of multiple layers of nanogratings that stack vertically, with potential applications in important fields such as photonic crystals.

### Refractive Index Sensor for Solvents

**Figure 3:**
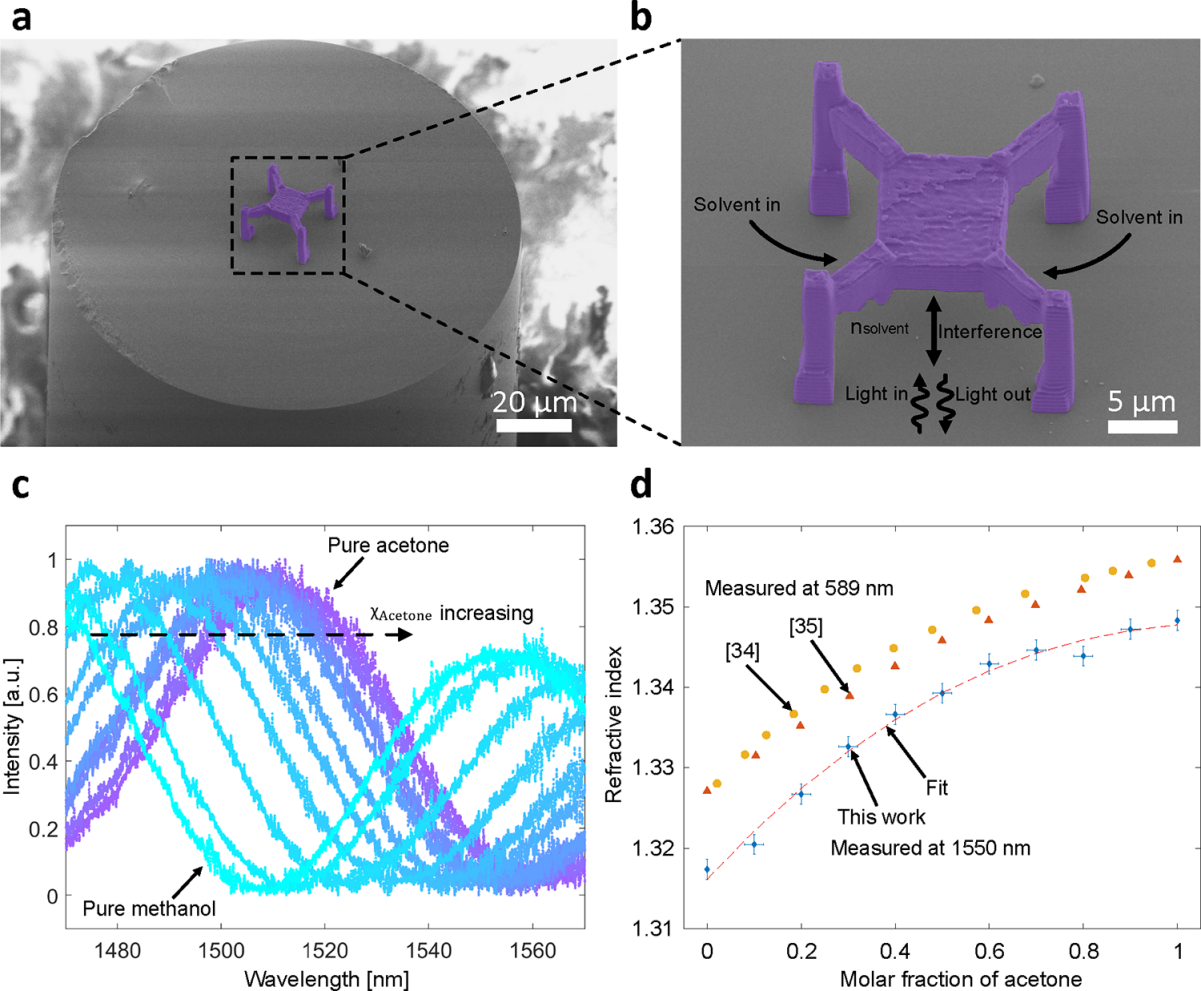
3D-printed refractive index sensor on the optical fiber tip. (a) Colored SEM image of the fiber-tip refractive index sensor. (b) An enlarged view illustrating the working principle of the fiber-tip refractive index sensor. (c) Normalized reflection spectra of the sensor immersed in binary mixtures of acetone and methanol at different molar fractions: from left to right, χ_acetone_ increases from 0 to 1. (d) Refractive index of the binary mixtures are plotted against χ_acetone_. The experimental data are fitted with a third-order polynomial model. Literature data measured at 589 nm are plotted for comparison.^34,35^

In contrast to 3D-printed polymers, our 3D-printed silica glass is resistant to common chemicals such as organic solvents, making it a good candidate for fabricating devices that operate in these scientifically and industrially important environments. Additionally, the smooth surface of the silica glass printed by using Uniform Mode is suitable for fabricating interferometric sensors requiring flat mirrors. Therefore, we used Uniform Mode to print a fiber-tip sensor for measuring the refractive index of organic solvents. See Methods for the detailed fabrication parameters. Figure 3a shows a colored SEM image of the refractive index sensor, and Figure 3b shows an enlarged view, illustrating the sensing principle. The sensor comprises a glass plate measuring 10 × 10 × 3.5 μm^3^ that is suspended 8.5 μm above the end face of the optical fiber, and four supporting posts extend 5 μm from the plate that facilitate the flow of solvents into the sensor. The combination of the glass plate and the optical fiber end face forms an open Fabry–Pérot interferometer. During the measurement, the liquid under measurement fills the open Fabry–Pérot cavity and, thus, changes the refractive index within it. The light injected from the fiber core interferes with the cavity, and a portion of the light will be reflected back to the fiber. If we use a broadband light source as an input and collect the reflection spectrum of the Fabry–Pérot interferometer, the wavelength of a dip of the interference pattern λ_*m*_ is given by
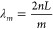
2where *n* is the refractive index of the medium within the cavity, *L* is the length of the cavity, and *m* is the order of the interference dip.^17^ If the length of the cavity remains unchanged during the measurement and we always track the same order of the interference dip, *L* and *m* are constant. By differentiating eq 2 with respect to *n*, we can get
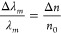
3where λ_*m*_ and *n*_0_ are the initial values of the wavelength of the interference dip and refractive index, respectively. Equation 3 indicates that the change of the refractive index is directly proportional to the wavelength shift of the interference dip. Using this principle, we can measure the refractive index change of a liquid sample by observing the optical response of the fiber-tip Fabry–Pérot interferometer.

We first evaluated the high-power stability of the sensor by coupling a laser operated at 1550 nm, followed by an optical amplifier, providing a maximum output power of over 100 mW to the fiber-tip sensor. We did not observe any structural deformation or detachment between the 3D-printed structure and the fiber tip after the high-power exposure. See the Methods section for details of the high-power exposure experiment.

The measurement setup is illustrated in Supporting Information, Figure S2, including a tunable laser that covers a spectral range between 1470 and 1570 nm, a fiber-optic circulator, and a wavelength domain component analyzer. We first used the refractive index sensor to measure sucrose solutions at different concentrations, and the reflection spectra are shown in the Supporting Information, Figure S3a. In Supporting Information, Figure S3b, the interference dip exhibits a linear red-shift as the refractive index increases, which shows a good agreement with eq 3. Benefiting from the excellent material properties of silica glass, our 3D-printed glass sensor neither swells nor deforms when immersed in organic solvents, as do most polymers. Therefore, we used it to measure the binary mixture of acetone and methanol, which cannot be done by using a polymer-based device. Figure 3c shows the reflection spectra of the sensor immersed in the binary mixtures of acetone and methanol, and from left to right, the acetone molar fraction χ_acetone_ increases from 0 to 1. Each spectrum was normalized to its maximum value. The reflection spectrum exhibits a red-shift as χ_acetone_ increases. Although Saunders et al. have reported the refractive index of pure acetone and methanol at 1550 nm,^33^ the refractive index of the binary mixture at this wavelength has not been reported previously. According to eq 3, we can use the refractive index values and the interference dips of the pure solvents to determine the refractive index values of the intermediate binary mixtures at different molar fractions, as shown in Figure 3d. Each data point represents an average of five measurements, and the experimental data are fitted with a third-order polynomial model with coefficients given in the Supporting Information, Table S2. The vertical error bars depict the root mean squared error (RMSE) of the fitted model. The horizontal error bars indicate the uncertainty in mixing to a particular molar fraction of the pure solvent (first and the last data points). Data from the literature measured at 589 nm using Abbe-type refractometers are plotted as well.^34,35^ Our measurements exhibit a similar trend with respect to the molar fraction compared to the literature data measured using 589 nm light sources, and the effects of chromatic dispersion result in lower absolute refractive index values. We note that due to the small volume of the cavity, we are sampling only a picoliter of solvent. In summary, we successfully demonstrated the refractive index measurement of aggressive organic solvents by using our 3D-printed glass refractive index sensor on the fiber tip. Furthermore, the excellent robustness of the 3D-printed sensor enabled us to perform multiple measurements without any notable structural deformation of the sensor.

### Polarization Beam Splitter

**Figure 4:**
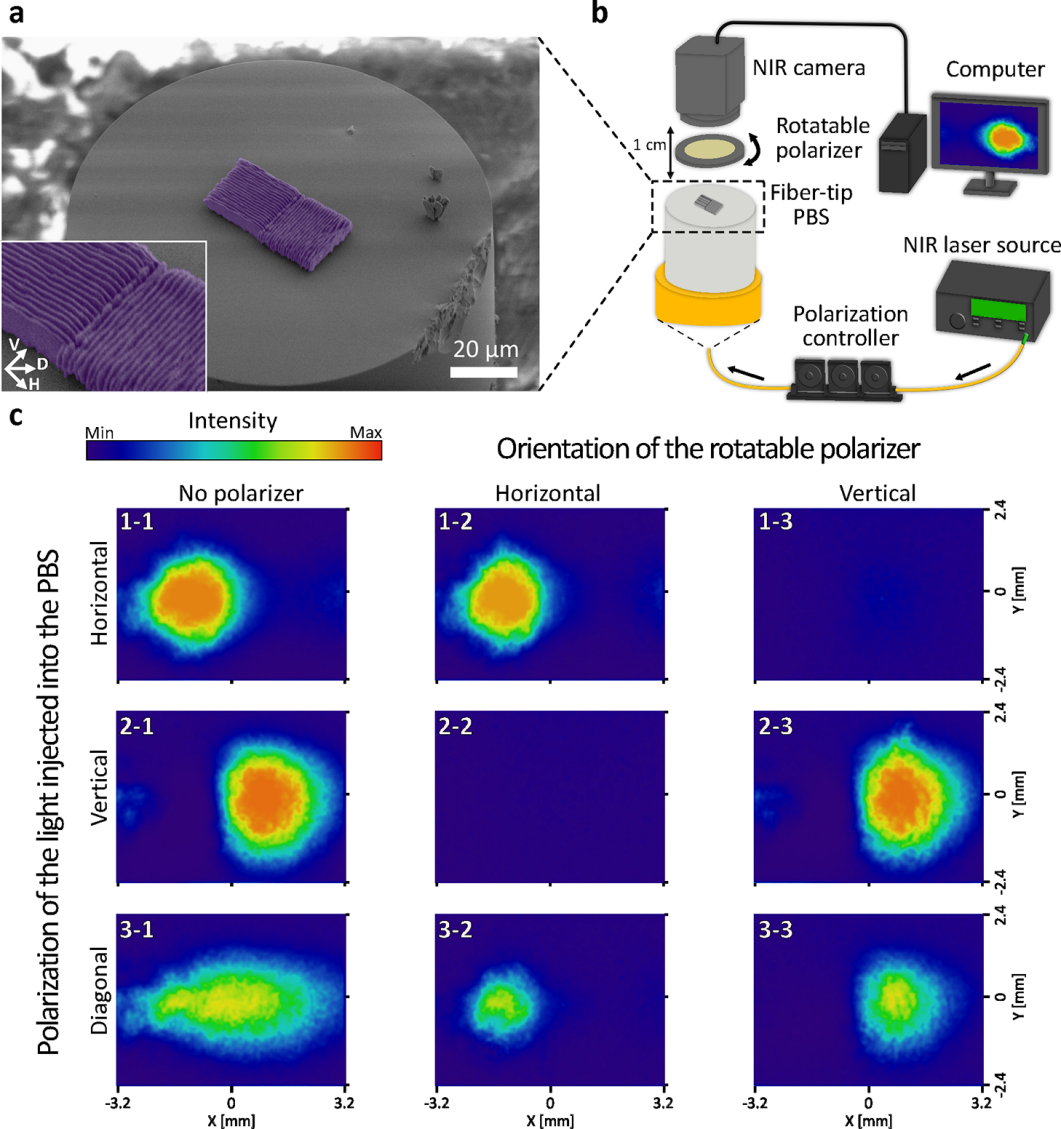
3D-printed PBS on the optical fiber tip. (a) The colored SEM image of the fiber-tip PBS. The inset is a close-up view of the PBS, and the arrows indicate its orientation. (b) The experimental setup. (c) Output beam profiles recorded by the NIR camera with different experimental settings. From the first to the third row, the polarization of the input light was set to horizontal, vertical, and diagonal with respect to the orientation of the fiber-tip PBS, respectively. The profiles in the first column were recorded without an analyzing polarizer. The profiles in the second and the third column were recorded with the analyzing polarizer’s transmission axis set horizontal and vertical to the orientation of the fiber-tip PBS, respectively. The color scale bar on the top left indicates the intensity of light.

PBSs are crucial optical components with widespread applications across fields, such as quantum photonics, optical communications, and spectroscopy. Subwavelength gratings (SWGs) have been used to create on-chip PBSs^36−38^ because such a 1D anisotropic metamaterial shows strong birefringence, making it an excellent choice for polarization manipulation. We integrated PBS on the fiber tip by using Nanograting Mode to print an SWG on the fiber tip. See Methods for the detailed fabrication parameters. Figure 4a shows a colored SEM image of the PBS, which consists of two SWG blocks with orthogonal grating orientations. Each grating has a period of 1.1 μm, the thickness of each nanoplate is 0.9 μm, and the height is 4.5 μm. The arrows in the inset indicate the orientation of the PBS. The experimental setup is illustrated in Figure 4b. We used a NIR laser diode with a central wavelength of 1550 nm and a paddle-style fiber-based polarization controller to generate arbitrarily polarized light. The polarized light was coupled to the fiber with PBS printed on the tip. We used an NIR camera placed 1 cm in front of the fiber tip to record the output beam profile from the PBS, and a rotatable linear polarizer can be placed between the PBS and the camera to determine the polarization orientation of the output beam. Figure 4c shows the output beam profiles recorded with the NIR camera. We first recorded the output profiles without the analyzing polarizer, as shown in the first column of Figure 4c. By setting the polarization orientation of the input light to horizontal or vertical with respect to the orientation of the fiber-tip PBS, we can steer the beam spot laterally through an angle of 12° (1–1 and 2–1). By setting the input light to diagonal polarization, we can create an elliptical beam spot at the center (3–1). Then we added the rotatable linear polarizer with an extinction ratio of 33 dB in front of the camera to study the polarization orientation of the beam spots. The beam profiles in the second and the third column of Figure 4c were recorded with the analyzing polarizer’s transmission axis set to horizontal and vertical with respect to the orientation of the fiber-tip PBS, respectively. The results in the first row (1–2 and 1–3) that show that the beam spot on the left side is horizontally polarized, and the results in the second row (2–2 and 2–3) show that the beam spot on the right side is vertically polarized. The elliptical spot in the third row is horizontally polarized on the left side and vertically polarized on the right side (3–2 and 3–3). To verify our experimental results, we used numerical simulation software to conduct finite difference time domain (FDTD) simulations. We created a structure identical to our fiber-tip PBS, injected light with different polarization orientations, and recorded the output beam profiles of the PBS, as shown in the Supporting Information, Figure S4. The simulated output magnitude profiles of the electric field with horizontally, vertically, and diagonally polarized input light are shown in Supporting Information, parts a, c, and e of Figure S5, respectively, which show similar E-field distributions to those in Figure 4c, 1–1, 2–1, and 3–1, respectively. In Supporting Information, Figure S5b,d, we can see that the main portion of the horizontally and vertically polarized input light is coupled to the grating with the same orientation as the polarization of the input light. This effect is due to the large birefringence of the anisotropic SWGs. In Figure S5f, the diagonally polarized input light is evenly transmitted through both gratings. See the Methods section for details of FDTD simulations.

The proposed fiber-tip PBS has potential application within hybrid integrated quantum photonic circuits.^39^ Specifically, in multichip integration setups, the fiber links chips operated in different environments, e.g., a quantum dot single-photon source operating at cryogenic temperatures and a photonic chip operating at room temperature. The fiber-tip PBS can select different polarization orientations of the photons emitted from the single-photon source and inject them into spatially separated waveguides on the photonic chip without any additional bulk optics.

## Conclusions

The process demonstrated in this work overcomes the limitations of other 3D DLW glass approaches that require high-temperature thermal treatment,^23−25^ enabling the fabrication of free-form glass structures on the tip of optical fibers with intact temperature-sensitive coatings and jackets. We measured the refractive index of the 3D-printed silica glass by spectral characterization of a fiber-tip glass cube Fabry–Perot etalon, thereby bridging a gap in our previous work.^26^ This information is crucial for designing and optimizing micro-optical components printed on fiber tips. Furthermore, these 3D-printed glass structures show exceptional resistance to many chemicals. We have demonstrated a fiber-tip refractive index sensor capable of robust measurement of the refractive index of aggressive organic solvents, in which polymer counterparts^16,17,20^ would suffer from swelling and deformation. The small sensor sampled a liquid volume of only a picoliter. Additionally, we report the refractive indices of mixtures of the common solvents acetone and methanol at near-infrared telecommunication wavelengths. These were not measured previously,^33^ and the data will help the calibration of emerging refractive index sensors operating within this important wavelength range. Finally, the demonstrated fiber-tip PBS enables the manipulation of light polarization and beam steering within an all-in-fiber system, which will find applications in fiber-to-chip coupling and integrated quantum photonic circuits.

Our work represents a significant step forward in photonics as it allows the printing of arbitrary optical glass components with subwavelength features directly onto the tips of optical glass fibers. It has potential in various fields such as fiber sensing, optical MEMS, and quantum photonics. For instance, the 3D-printed glass fiber sensor could be integrated into microfluidic devices to measure fluid flow and monitor the concentration of organic solvents.^40^ Additionally, a MEMS accelerometer consisting of suspended cantilevers and proof mass^41^ could be printed on the fiber tip, enabling the measurement of proof mass displacement through optical readouts. Finally, fiber-integrated quantum emitters could be enabled by transferring two-dimensional (2D) materials on the fiber tips, followed by patterning the 2D materials using femtosecond laser^42^ and 3D printing of glass microlenses on the 2D materials to improve coupling efficiency, thereby addressing on the main limitations of current single photon emitters.

## Methods

### Sample Preparation

The SMF-28 single-mode optical glass fiber was acquired from Thorlabs. It has a 10.4 μm mode field diameter at 1550 nm, 0.14 NA, and a 125 μm cladding diameter. We cleaved the fiber with a standard fiber cleaver and removed the Hytrel outer jackets and acrylate coating close to the end of the fiber, leaving a 1 cm segment of glass fiber uncovered. We inserted the fiber tip through the customized fiber holder, which was made of two parts. The top part of the fiber holder was made of plastic, fabricated by a commercial 3D printer (Form 3, Formlabs, USA). It comprises a 0.2 mm hole that keeps the fiber tip from moving and a flat top surface that allows the HSQ solution to form a dome shape on it. The bottom part of the holder was an aluminum CNC machined part, which consists of a joint that can assemble with the top part of the holder and screw holes at the bottom that can be fixed firmly to the motorized stage in the femtosecond laser workstation. We drop-casted the HSQ solution on the fiber tip using a micropipet: two droplets of HSQ solution with the volume of 0.3 μL and 1.0 μL were drop-casted, subsequently. The HSQ solution was comprised of HSQ powder (H-SiOx, Applied Quantum Materials Inc., Canada) dissolved in toluene (Sigma-Aldrich, USA). We also tried the HSQ solution that contained HSQ powder dissolved in methyl isobutyl ketone, and we found that HSQ powder dissolved in toluene resulted in more controlled differentiation between the two printing modes. Finally, we let the solvent evaporate in ambient conditions for more than 24 h before DLW.

### Direct Laser Writing

The femtosecond laser workstation consists of a high-power femtosecond laser source (Spirit 1040-4-SHG, Spectra-Physics of Newport, Newport, CT, USA), a linear motorized stage (XMS100, Newport, USA), and a CCD camera with LED illumination. We fixed the sample to the motorized stage with four screws. We used a 40× microscope objective (Plan Achromat RMS40X, Olympus, Japan) to focus the laser on the sample and obtain the real-time image via the CCD camera. The energies of the laser pulses were measured with a silicon optical power detector (918D-SL-OD3R, Newport, USA) after the pulses exited the objective. We located the core of the fiber by injecting a low-power visible laser from the other end of the fiber, and the core was illuminated and seen with the camera. In order to find the interface between the HSQ layer and the end face of the optical fiber, we increased the laser power to 1.5 times the typical power for writing, focused the laser below the end face of the fiber tip, and gradually decreased the sample until we saw a shining plasma on the camera, which indicated the interface was reached. We repeated this interface-finding process at three different locations around the fiber core and recorded their coordinates. These three coordinates allowed us to correct the tilt angle of the fiber tip. We started the DLW 0.5 μm below the interface to ensure that the 3D-printed structure has robust contact with the end face of the optical fiber.

### Development Process

We used a basic developer to remove the unexposed HSQ and release the 3D-printed structure on the fiber tip. The developer was a 0.1 M potassium hydroxide (Sigma-Aldrich, USA) in deionized water mixed with 0.05 vol % of Triton X-100 (LabChem, USA), which was added as a surfactant to decrease the size of bubbles formed during the development process and prevent the 3D-printed structure from being damaged by bubbles. The development was done using a customized development setup, which included a hollow cylinder that can be assembled with the top part of the fiber holder and two small containers connected to the hollow cylinder by channels. It allowed us to fill the developer into the small containers, and the developer flowed through the channels to the cylinder and gently submerged the HSQ layer to avoid damaging the 3D-printed structure on the fiber tip. The development process took 1 h, and the sample was left to dry in ambient conditions.

### Fitting of the Reflection Spectrum of the Glass Cube

The measured reflection spectra of the 3D-printed glass cube were analyzed by fitting with a sum of sines model to allow us to extract the FSR of the Fabry–Pérot etalon. Each reflection spectrum was fitted to the model function
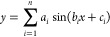
4where *a*_*i*_ is the amplitude, *b*_*i*_ is the frequency, *c*_*i*_ is the phase constant for each sine wave term, and *n* is the number of terms. We selected *n* = 3 as the number of terms and fitted the spectra in the wavelength range between 1470 and 1570 nm. The fitting algorithm used was the genetic algorithm from Matlab’s Curve Fitting Toolbox (Matlab R2022b, USA).

### Optical Profilometer Measurement

We used an optical profilometer (Wyko NT9300, Veeco, USA) to measure the height of the 3D-printed glass cube. A 20× objective was used that provided an in-plane resolution of 0.67 μm. We used the vertical scanning interferometry (VSI) mode that uses the short coherence length of white light to measure the degree of fringe modulation (coherence) between the reference and sample beams while the objective moves vertically through the sample. The vertical position of the objective at peak fringe contrast for each point on the sample surface was then recorded to generate a topographical map of the sample surface. The minimum vertical resolution of the VSI mode is 3 nm.

### Calculation of Group Refractive Index

We used the following equation to calculate the group index:
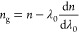
5where λ_0_ is the wavelength of light in vacuum.^28^ The dispersion equation of fused silica is
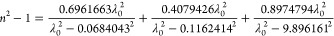
6where λ_0_ is expressed in micrometer.^29^ We calculated *n*_g_ = 1.4621 at λ_0_ = 1.47 μm, and *n*_g_ = 1.4627 at λ_0_ = 1.57 μm.

### Printing the Refractive Index Sensor

We printed the fiber-tip refractive index sensor using Uniform Mode. The laser power was 0.3 mW, and the repetition rate was 10 kHz. The printing time was around 4 h, and the development time was 2 h.

### High-Power Exposure Experiment

We connected the fiber of the refractive index sensor to a laser operated at 1550 nm (TSL-570, Santec, Japan), followed by an optical amplifier (EDFA100P, Thorlabs, USA), which provides a maximum output power of over 20 dBm (100 mW). During the test, the power was kept constant at the highest available power for at least 1 min.

### Printing the Polarization Beam Splitter

We printed the fiber-tip PBS using the Nanograting Mode. The laser power was 2.5 mW, and the repetition rate was 50 kHz. The printing time was less than 10 min, and the development time was 2 h.

### FDTD Simulations

The simulations were conducted using an Ansys Lumerical FDTD solver. We created a structure identical to our 3D-printed PBS in the software, including two sets of grating with a period of 1.1 μm and a thickness of 0.9 μm. The gratings have orthogonal orientations, and each covers half of the fiber core, as shown in Supporting Information, Figure S4. We injected a guided fundamental mode source from the optical fiber into the PBS structure, and we can control the polarization orientation of the mode source with respect to the orientation of the PBS. We placed E-field profile monitors across and along the propagation axis of the fiber to record the top view and side view of the E-field profile, respectively, and the results are shown in Supporting Information, Figure S5.

## Supporting Information Available

Reflection spectra in different environments, refractive index estimation, measurement setup for the fiber-tip refractive index sensor, refractive index measurements in sucrose solutions, coefficients of the third order polynomial model in Figure 3d, setup for FDTD simulations, and simulated magnitude profiles of the electric field (PDF)
