# Supplementary material for: 3D Printing of Glass Micro-Optics with Subwavelength Features on Optical Fiber Tips
Source: ACS Nano. 2024 Mar 29;18(16):10788–97. doi: 10.1021/acsnano.3c11030 (PMC11044591; doi:10.1021/acsnano.3c11030)
Supplement: Supplementary file 1 — nn3c11030_si_001.pdf [file nn3c11030_si_001.pdf]

Supporting Information

3D Printing of Glass Micro-Optics with Subwavelength Features on Optical Fiber Tips

Lee-Lun Lai,[†,‡] Po-Han Huang,[†,‡] Göran Stemme,[†] Frank Niklaus,[†] and Kristinn B. Gylfason[*,†]

†*Division of Micro and Nanosystems, School of Electrical Engineering and Computer Science, KTH Royal Institute of Technology, Stockholm 10044, Sweden*

‡*These authors contributed equally to this work*

E-mail: gylfason@kth.se

Phone: +46 8 790 9231

The Supporting Information includes:

Supporting Figures 1-5

Supporting Table 1-2

References

Glass Cube for Refractive Index Estimation

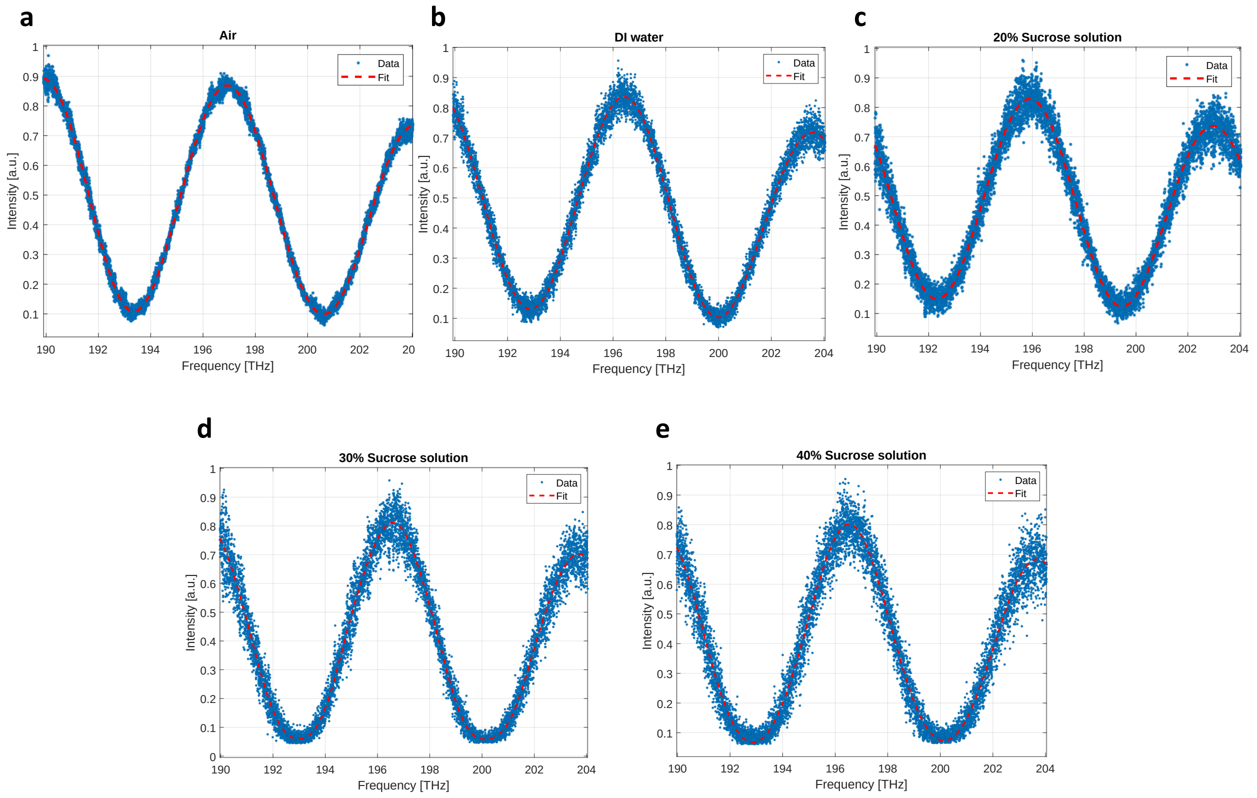

Figure. S1: Reflection spectra and fitted curves of the fiber-tip glass cube in different environments. **a** In the air. **b** In the deionized water. **c** In the 20% sucrose solution. **d** In the 30% sucrose solution. **e** In the 40% sucrose solution.

Table. S1: Group index measured from different environments where the fiber-tip cube was placed in.

Environment	Measured RI
Air	1.46
Deionized water	1.48
20% Sucrose solution	1.48
30% Sucrose solution	1.48
40% Sucrose solution	1.46
50% Sucrose solution	1.47
Average	1.47

Refractive Index Sensor

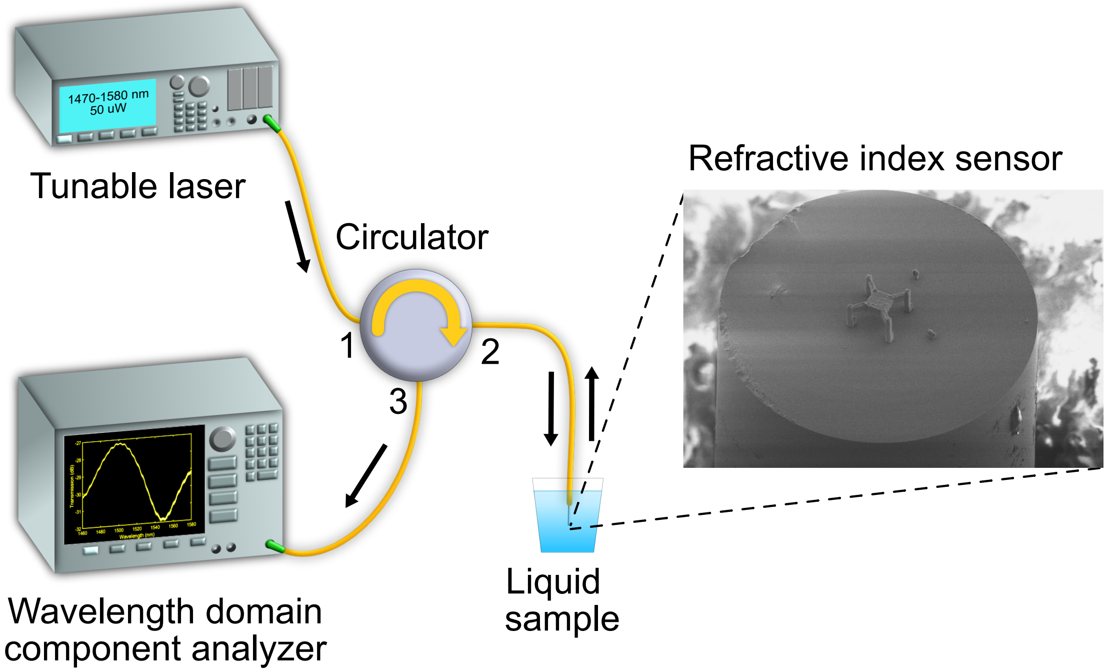

Figure. S2: The measurement setup for the fiber-tip refractive index sensor.

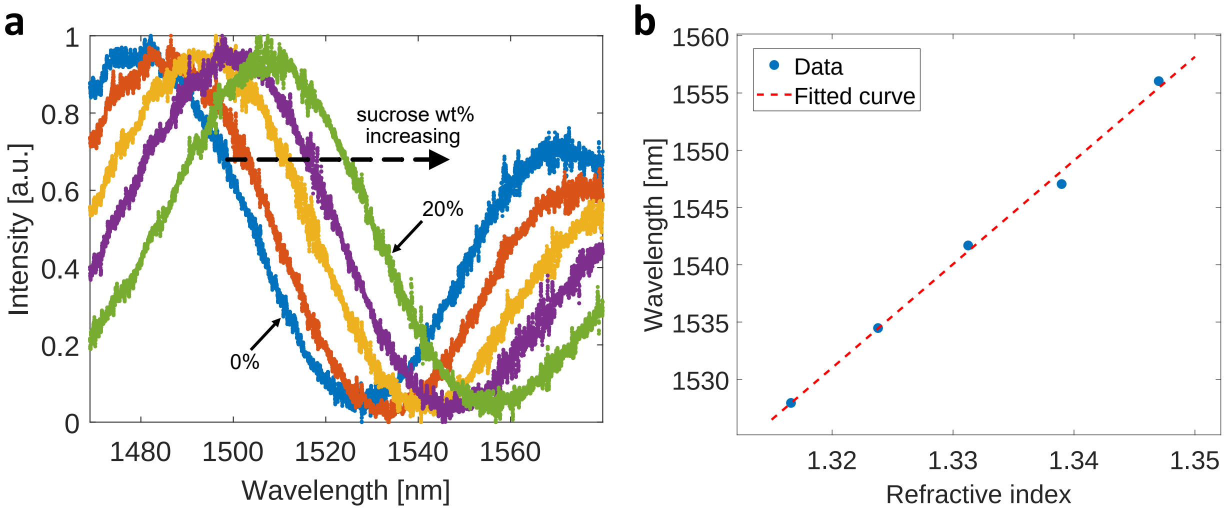

Figure. S3: The refractive index measurements in sucrose solutions. **a** The spectra show the normalized reflection intensity of the sensor immersed in the sucrose solutions at different concentrations (wt%): 0%, 5%, 10%, 15%, and 20%. **b** The refractive index of each sucrose solution at 1550 nm can be calculated using a third-order polynomial model reported in a prior study.[1] The wavelengths of the resonance dips are plotted against the refractive index of the sugar solutions. Experimental data are fitted with a first-order polynomial model.

Table. S2: Coefficients of the third order polynomial model for predicting the refractive index of the binary mixtures of acetone and methanol at different acetone molar fractions χ_A using a near-infrared light source in the wavelength range 1470 – 1570 nm.

$$n = c_1\chi_A^3 + c_2\chi_A^2 + c_3\chi_A + c_4$$

c_1	c_2	c_3	c_4
0.005061	-0.03686	0.06335	1.316

Polarization Beam Splitter

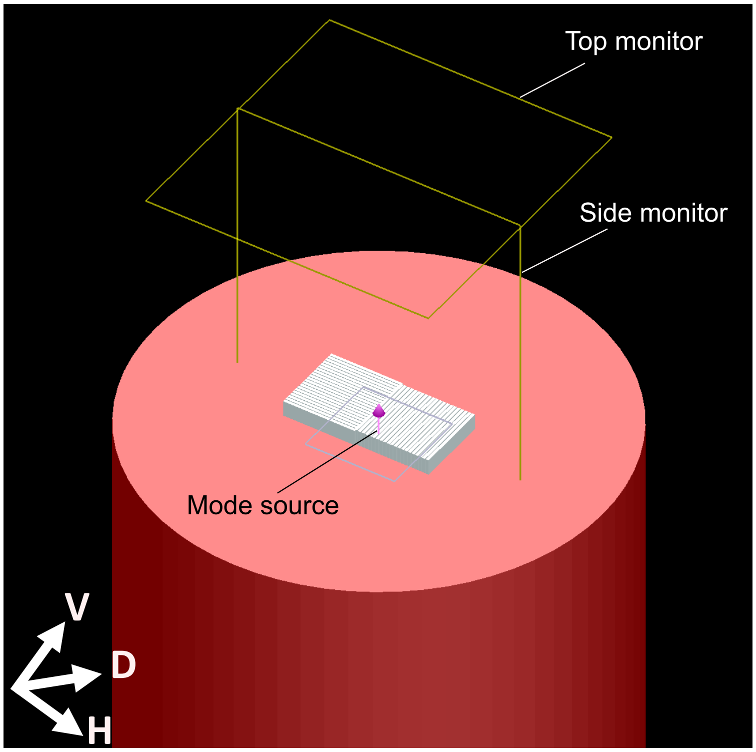

Figure. S4: Setup for finite difference time domain simulation. The mode source was selected to fundamental mode guided in the optical fiber, and the polarization of light with respect to the orientation of the PBS can be controlled. The top monitor located 100 µm above the fiber tip recorded the E-field magnitude profile across the propagation axis of the fiber. The side monitor recorded the cross-section E-field magnitude profile along the horizontal axis of the PBS. The arrows on the bottom left indicate the orientation of the 3D-printed PBS.

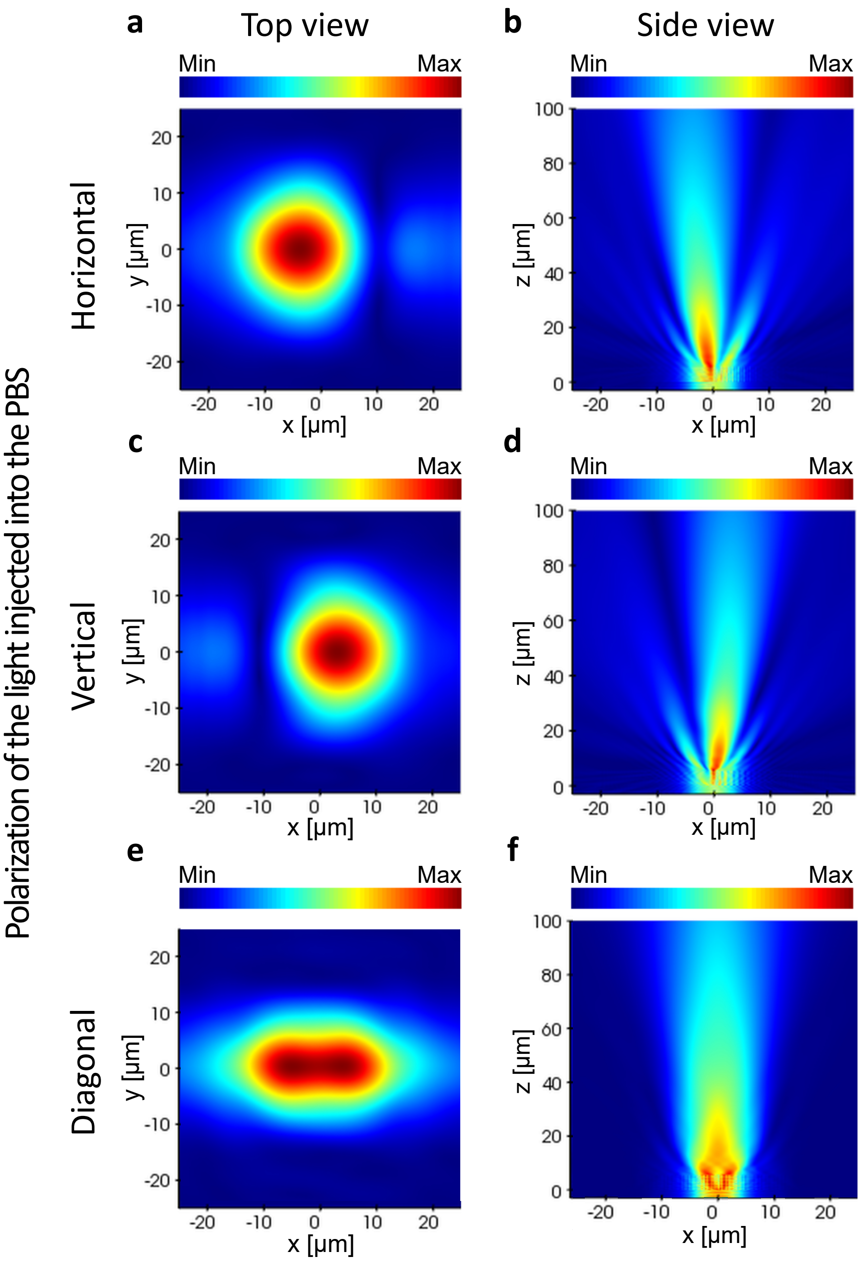

Figure. S5: Simulated magnitude profiles of the electric field of the top view and side view of the fiber-tip PBS with the input light set to different polarization orientations. **a**, **b** Horizontally polarized light. **c**, **d** Vertically polarized light. **e**, **f** Diagonally polarized light.

References

(1) Saunders, J. E.; Sanders, C.; Chen, H.; Loock, H.-P. Refractive Indices of Common Solvents and Solutions at 1550 nm. *Applied Optics* **2016**, *55*, 947.
